# Genetic Characterization of Clade 2.3.2.1 Avian Influenza A(H5N1) Viruses, Indonesia, 2012

**DOI:** 10.3201/eid2004.130517

**Published:** 2014-04

**Authors:** Ni Luh Putu Indi Dharmayanti, Risza Hartawan, Pudjiatmoko, Hendra Wibawa, Hardiman, Amanda Balish, Ruben Donis, C. Todd Davis, Gina Samaan

**Affiliations:** Indonesian Research Center for Veterinary Science, Bogor, Indonesia (N.L.P.I. Dharmayanti, R. Hartawan, Hardiman);; Ministry of Agriculture, Jakarta, Indonesia (Pudjiatmoko);; Disease Investigation Center Wates, Jogjakarta, Indonesia (H. Wibawa);; Centers for Disease Control and Prevention, Atlanta, Georgia, USA (A. Balish, R. Donis, C.T. Davis);; Centers for Disease Control and Prevention, Jakarta (G. Samaan)

**Keywords:** orthomyxovirus, highly pathogenic avian influenza virus, H5N1, phylogenetics, Indonesia, evolution, outbreak, surveillance, viral, geographic distribution, viruses, influenza virus, zoonoses

## Abstract

After reports of unusually high mortality rates among ducks on farms in Java Island, Indonesia, in September 2012, influenza A(H5N1) viruses were detected and characterized. Sequence analyses revealed all genes clustered with contemporary clade 2.3.2.1 viruses, rather than enzootic clade 2.1.3 viruses, indicating the introduction of an exotic H5N1 clade into Indonesia.

Highly pathogenic avian influenza A(H5N1) virus has circulated in poultry in Indonesia since 2003 (*1*,*2*). The phylogeny of A(H5N1) viruses detected during 2003–2011 indicated all genes descended from 1 ancestral virus with a clade 2.1 hemagglutinin (HA) introduced into Indonesia before 2003 (*3*). These viruses became enzootic and evolved into second-, third-, and fourth-order HA clades, leading to the recent dominance of clade 2.1.3.2 viruses (*4*). Outbreaks in poultry typically caused high mortality rates among gallinaceous birds, especially layer, broiler, and native chickens. The virus seemed less pathogenic in aquatic birds (*5*). However, reports of duck deaths and a higher than usual mortality rate (100% in some outbreaks) in backyard farms in Central Java, Jogjakarta, and East Java Provinces, Indonesia, in September 2012 triggered a joint outbreak investigation by animal and public health authorities (*6*). We describe the genetic characteristics of viruses isolated from A(H5N1) infection outbreaks in these 3 provinces on Java Island, where a previously unrecognized clade was detected.

## The Study

We investigated 9 small-holding duck farms that reported bird deaths during September 12–November 5, 2012 (*6*). Cloacal swab samples were collected from sick birds, placed in 1,000 µL of viral transport medium, and sent for testing at laboratories of the regional Ministry of Agriculture Disease Investigation Center, Jogjakarta. Seventeen A(H5N1)–positive samples were forwarded to the National Animal Health Laboratory, Indonesian Research Center for Veterinary Science (IRCVS), for virus isolation and genome sequencing. 

In addition, IRCVS collected 122 cloacal swab samples from birds and 58 environmental swab samples (from defeathering machines) at 5 live-bird markets (LBMs) in East Java Province during November 5–8, 2012. RNA extracted from farm and LBM specimens was tested for influenza A matrix gene to identify presumptive A(H5N1)-positive samples (*7*). Select positive samples were inoculated in 9–11-day-old embryonated, specific pathogen–free eggs. Allantoic fluid was harvested 36 h postinfection and tested for HA with chicken erythrocytes to confirm virus isolation (*8*). 

Samples showing suspected A(H5N1) infection were propagated in a Biosafety Level-3 laboratory at IRCVS in compliance with biosafety regulations. Ten virus isolates (7 from duck farms, 3 from LBMs) were chosen for full-length HA gene sequencing (GenBank accession nos. KC417271–KC417277, KC757643); 4 were selected for genome sequencing. Results of reverse transcription PCR and sequencing primers are available on request. Sequencing and consensus sequence generation were conducted as described (*9*). Phylogenetic trees were generated by using MEGA4 (*10*) (Figure; Technical Appendix 1).

**Figure:**
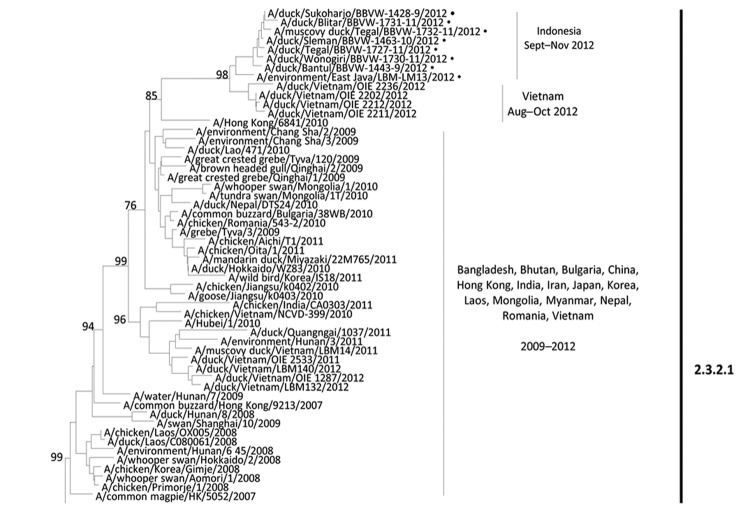
Partial phylogenetic tree of influenza A(H5N1) hemagglutinin (HA) gene sequences. The phylogenetic tree was generated in MEGA version 4 (www.megasoftware.net), using neighbor-joining analysis with 1,000 bootstrap replicates and the Kimura 2-parameter model. Viruses characterized in this study are indicated with a dot. The HA tree was rooted to A/goose/Guangdong/1/1996.

Phylogenetic analysis revealed that A(H5N1) isolates from samples collected from duck farm outbreaks and an LBM were not related to isolates in long-established Indonesian clade 2.1; rather, the HA genes closely resembled those of clade 2.3.2.1 viruses recently found in Vietnam, China, and Hong Kong (Figure). Full-length HA genes showed 97%–98%-nt identity with recent viruses from Vietnam and clustered in a larger group containing viruses from many Asian regions during 2009–2012. The environmental sample from an East Java LBM shared >99% nt similarity with viruses from samples at duck farms, indicating spread of this A(H5N1) clade into the marketing chain. A poultry sample from the same district as the 2.3.2.1 virus was identified as clade 2.1.3.2 (Figure), indicating likely cocirculation. 

The 8 clade 2.3.2.1 HA genes analyzed possessed a multibasic amino acid cleavage site (Table 1). The cleavage site sequence of the clade 2.3.2.1 viruses from Indonesia (PQRE^del^RRRKR↓G) differed from recent clade 2.1.3.2 viruses (PQRE**S**RR**K**KR↓G) by a Ser deletion at position 325 and a K328R substitution. Like other serotype H5N1 HA proteins, all isolates possessed a conserved glutamine at position 222 (equivalent to H3 position 226) and glycine at position 224 (H3 position 228), indicating no substantial changes in avian receptor-binding specificity (Table 1) (*11*). The clade 2.3.2.1 viruses from Indonesia possessed 6 or 7 potential N-linked glycosylation sites (7 in clade 2.1.3.2 viruses), but unlike 2.1.3.2 viruses, all 2.3.2.1 viruses lacked the potential glycosylation site at position 154. 

**Table 1:** Genetic characteristics of influenza A(H5N1) clade 2.3.2.1 viruses found in Indonesia, 2012*†

Strain name	PB2			PB1-F2			HA‡				NA		M2			NS sequence	
	aa 627	aa 701		PB1-F2 truncation	aa 66		aa 222	aa 224	Cleavage site		aa 203		aa 27	aa 31		aa 80–84 del	PDZ binding ligand
A/Hubei/1/2010	E	D		90 aa	N		Q	G	PQRERRRKR↓G		I		I	S		Yes	ESEV
A/Hong Kong/ 6841/2010	E	D		90 aa	N		Q	G	PQRERRRKR↓G		I		I	S		Yes	ESEV
A/env/East Java/ LBM-LM13/2012	E	D		57 aa	N		Q	G	PQRERRRKR↓G		V		I	S		Yes	ESEV
A/duck/Sukoharjo/ BBVW-1428–9/2012	E	D		57 aa	N		Q	G	PQRERRRKR↓G		I		I	S		Yes	ESEV
A/duck/Bantul/ BBVW-1443-9/2012	E	D		57 aa	N		Q	G	PQRERRRKR↓G		I		I	S		Yes	ESEV
A/duck/Sleman/ BBVW-1463–10/2012	E	D		57 aa	N		Q	G	PQRERRRKR↓G		ND		I	S		Yes	ESEV
A/md/Tegal/ BBVW-1732-11/2012	ND	ND		ND	ND		Q	G	PQRERRRKR↓G		ND		ND	ND		ND	ND
A/dk/Blitar/ BBVW-1731-11/2012	ND	ND		ND	ND		Q	G	PQRERRRKR↓G		ND		ND	ND		ND	ND
A/dk/Tegal/ BBVW-1727-11/2012	ND	ND		ND	ND		Q	G	PQRERRRKR↓G		ND		ND	ND		ND	ND
A/dk/Wonogiri/ BBVW-1730-11/2012	ND	ND		ND	ND		Q	G	PQRERRRKR↓G		ND		ND	ND		ND	ND

*HA, hemagluttinen; NA, neuraminidase; M2, matrix 2; NS, nonstructural; ND, not determined. †Numbering of the first and last nucleotide position of the gene that was sequenced is as follows: PB2, 1618–2192; PB1, 130–632; PA, 34–429; HA, 1–1710; NP, 268–755; NA, 55–1314; M, 55–919; NS, 25–690. ‡Glycosylation motif at 154 was absent for all strains.

Up to 29 conserved amino acid changes occurred in the mature HA1 protein between clade 2.3.2.1 and clade 2.1.3.2 viruses found recently in Indonesia, indicating these A(H5N1) virus subgroups probably diverged substantially in antigenicity. In contrast, the HA1 of the new viruses collected in Indonesia differed by 8–10 aa from A/Hubei/1/2010, the most closely related clade 2.3.2.1 A(H5N1) candidate vaccine virus recommended by the World Health Organization (Technical Appendix 2) (*12*). 

To test the antigenic relationship of the clade 2.3.2.1 virus to the endemic clade 2.1.3.2 virus, we conducted a hemagglutination-inhibition test with ferret antiserum raised against viruses from these and other H5N1 clades (Table 2) (*8*). As the HA1 protein sequence differences suggest, clade 2.1.3.2 antiserum did not inhibit hemagglutination by a representative clade 2.3.2.1 virus from Indonesia, A/environment/East Java/LBM-LM13/2012. In contrast, this virus cross-reacted with antiserum to clade 2.3.2.1 viruses from other countries at heterologous titers generally within 2-fold of or equivalent to the homologous virus titer. The Indonesian clade 2.3.2.1 virus was most closely related antigenically to viruses that clustered genetically into the A/Hong Kong/6841/2010-like group of clade 2.3.2.1 (Table 2).

**Table 2:** Hemagglutination-inhibition assay of clade 2.3.2.1 highly pathogenic avian influenza A(H5N1) virus introduced into Indonesia, 2012*

Antigen†	Clade	Reference ferret antiserum								
		1	2.2.1	2.3.4	2.1.3.2	2.3.2.1	2.3.2.1	2.3.2.1	2.3.2.1	2.3.2.1
		VN/1203	EG/321	ANH/1	IND/ 12379	CH/1 RG30	BS/HK/ 1161	BHG/MG/X53	HK/ 6841	DK/VN/ 1584
Reference strains										
VN/1203	1	320*	20	40	<10	10	<10	10	10	10
EG/321	2.2.1	80	**1,280**	80	20	40	10	80	80	40
ANH/1	2.3.4	160	80	**640**	80	<10	<10	<10	40	10
IND/12379	2.1.3.2	10	10	40	**1,280**	<10	<10	<10	40	40
CH/1 RG30	2.3.2.1	40	80	20	20	**640**	40	160	640	160
BS/HK/1161	2.3.2.1	<10	40	10	<10	320	**80**	160	640	80
BHG/MG/X53	2.3.2.1	10	80	20	20	320	40	**320**	640	320
HK/6841	2.3.2.1	10	20	10	20	160	20	320	**640**	160
DK/VN/1584	2.3.2.1	<10	40	10	20	320	20	320	320	**160**
Test strain										
A/environment/East Java/LBM-LM13/2012	2.3.2.1	<10	40	10	10	160	40	320	320	160

*Homologous titers of reference antigen to ferret antiserum are indicated in boldface. †Strains: VN/1203, A/Vietnam/1203/2004; EG/321, A/Egypt/2321-NAMRU3/2007; ANH/1, A/Anhui/1/2005; IND/12379, A/Indonesia/NIHRD12379/2012; CH/1 RG30, A/Hubei/1/2010 IDCDC-RG30; BS/HK/1161, A/barn swallow/Hong Kong/1161/2010; BHG/MG/X53, A/barheaded goose/Mongolia/X53/2009; HK/6841, A/Hong Kong/6841/2010; DK/VN/1584, A/duck/Vietnam/NCVD-1584/2012.

All 4 isolates exhibited the typical 20-aa deletion in the stalk region (residue 48–68) of the neuraminidase gene (NA). Although 1 sample had an Ile203Val substitution in the NA, which has been associated with reduced susceptibility to oseltamivir, no other markers of resistance in the NA or M2 were identified (Table 1). All 4 viruses had NS1 protein sequences with the typical deletion at position 80–84 and an intact H5N1 consensus PDZ binding motif (ESEV). A truncated form (57 aa) of the PB1-F2 protein was found in all viruses characterized. Although the functional consequences of this truncation are unknown, this represents a change from the typical full-length 90-aa protein found in most A(H5N1) viruses (*13*). All other amino acid residues and motifs of interest in the internal genes of the 4 viruses sequenced in this study represented avian consensus sequences. 

Phylogenetic comparison of the NA and internal gene segments revealed ancestral origins of the new viruses similar to those of the HA gene (Technical Appendix 1). Although partial nucleotide sequences from some genes were available for analysis (Table 1), sequence identities and phylogenetic comparisons to other clade 2.3.2.1 genomes in GenBank and Global Initiative on Sharing Avian Influenza Data databases confirmed their relatedness to viruses circulating recently in China, Vietnam, and Hong Kong. Individual gene sequence analysis did not show reassortment between these clade 2.3.2.1 viruses and the previously identified clade 2.1.3.2 genotype virus in Indonesia.

## Conclusions

Detection of a novel clade of A(H5N1) virus in Indonesia marks a potential turning point in the molecular epidemiology of this virus. Indonesia has the highest number of human A(H5N1) infections because of ongoing outbreaks in poultry (*14*,*15*). Whether this new virus will become entrenched, as did clade 2.1.3 viruses over the past decade, remains to be seen, as do its effects on the incidence of human infection. Potential cocirculation of subtypes of 2 different clades warrants review of diagnostic methods and vaccination strategy to maximize effectiveness of disease control interventions. The lack of antigenic relatedness between the clade 2.3.2.1 and 2.1.3.2 viruses must be considered when evaluating A(H5N1) serologic diagnostic reagents used in Indonesia. This change also may have implications in selecting prepandemic candidate vaccine virus for the region. Furthermore, poultry vaccines may need to be matched antigenically to circulating virus if clade 2.3.2.1 virus continues to circulate in Indonesia. Introduction of this virus is a stark reminder of the value of control measures to reduce the spread of subtype H5N1 and the need for enhanced surveillance of humans and poultry to monitor changes in its genetic and immunologic features.

Technical Appendix 1 A–G. Phylogenetic tree of PB2, PB1, PA, NP, NA, M, NS (A = PB2; B = PB1; C = PA; D = NP; E = NA; F = M; G = NS). The phylogenetic tree was generated in MEGA version 4, using neighbor-joining analysis with 1000 bootstrap replicates using the Kimura-2 parameter model. Viruses characterized in this study are indicated with a bar showing samples collected in Indonesia from September to November 2012.

Technical Appendix 2 Phylogenetic tree of PB2, PB1, PA, NP, NA, M, NS (A = PB2; B = PB1; C = PA; D = NP; E = NA; F = M; G = NS). From bird samples collected in Indonesia during September–November 2012.
