# Supplementary material for: Genetic Characterization of Clade 2.3.2.1 Avian Influenza A(H5N1) Viruses, Indonesia, 2012
Source: Emerg Infect Dis. 2014 Apr;20(4):671–4. doi: 10.3201/eid2004.130517 (PMC3966381; doi:10.3201/eid2004.130517)
Supplement: Technical Appendix 1 — A–G. Phylogenetic tree of PB2, PB1, PA, NP, NA, M, NS (A = PB2; B = PB1; C = PA; D = NP; E = NA; F = M; G = NS). The phylogenetic tree was generated in MEGA version 4, using neighbor-joining analysis with 1000 bootstrap replicates using the Kimura-2 parameter model. Viruses characterized in this study are indicated with a bar showing samples collected in Indonesia from September to November 2012. [file 13-0517-Techapp-s1.pdf]

DOI: http://dx.doi.org/10.3201/eid2004.130517

Genetic Characterization of Clade 2.3.2.1 Avian Influenza A(H5N1) Viruses, Indonesia, 2012

Ni Luh Putu Indi Dharmayanti, Risza Hartawan, Pudjiatmoko, Hendra Wibawa, Hardiman, Amanda Balish, Ruben Donis, C. Todd Davis, and Gina Samaan

Technical Appendix 1.

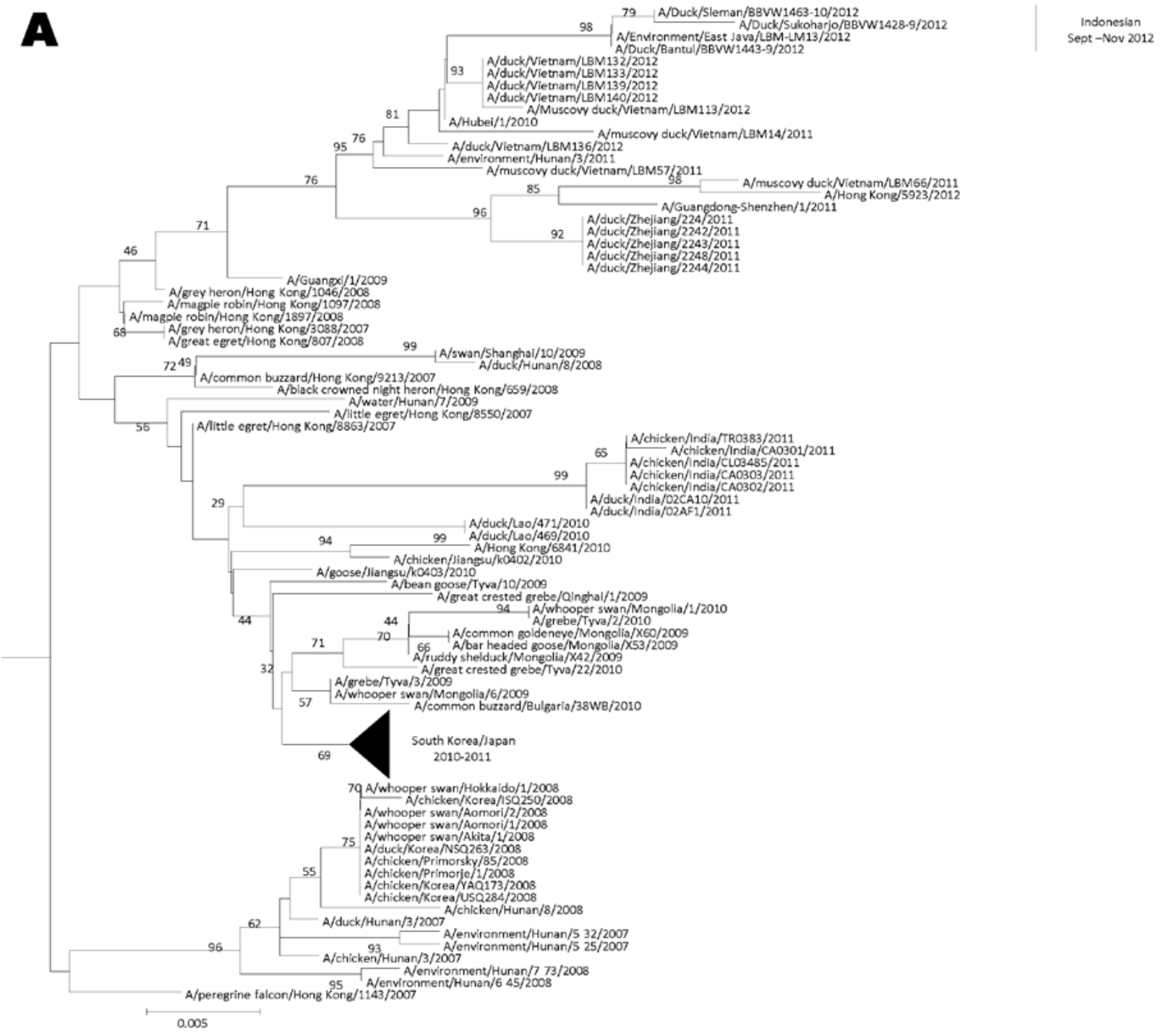

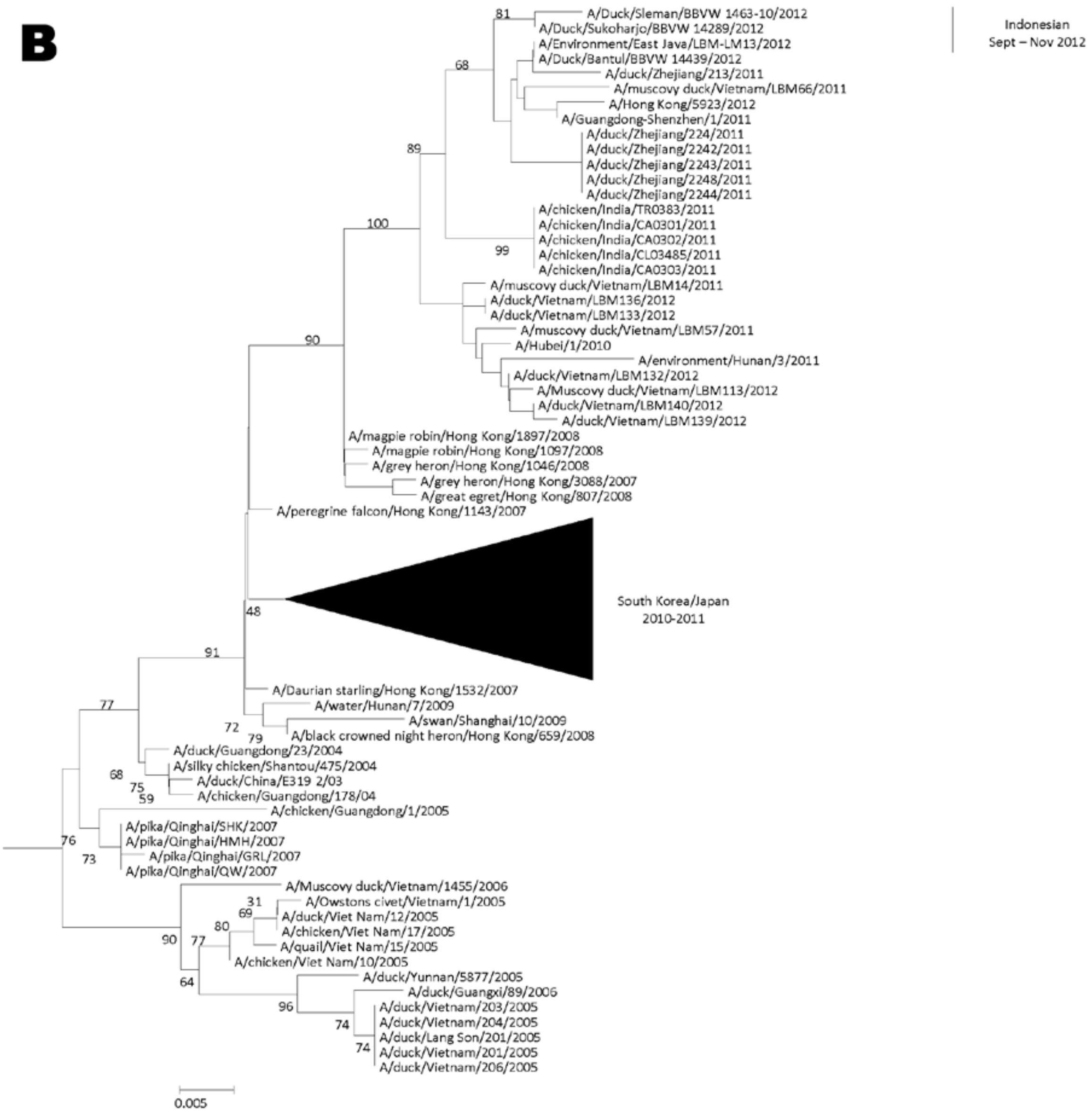
B
A/Duck/Sleman/BBVW 1463-10/2012
A/Duck/Sukoharjo/BBVW 14289/2012
A/Environment/East Java/LBM-LM13/2012
A/Duck/Bantul/BBVW 14439/2012
A/duck/Zhejiang/213/2011
A/muscovy duck/Vietnam/LBM66/2011
A/Hong Kong/5923/2012
A/Guangdong-Shenzhen/1/2011
A/duck/Zhejiang/224/2011
A/duck/Zhejiang/2242/2011
A/duck/Zhejiang/2243/2011
A/duck/Zhejiang/2248/2011
A/duck/Zhejiang/2244/2011
A/chicken/India/TR0383/2011
A/chicken/India/CA0301/2011
A/chicken/India/CA0302/2011
A/chicken/India/CL03485/2011
A/chicken/India/CA0303/2011
A/muscovy duck/Vietnam/LBM14/2011
A/duck/Vietnam/LBM136/2012
A/duck/Vietnam/LBM133/2012
A/muscovy duck/Vietnam/LBM57/2011
A/Hubei/1/2010
A/environment/Hunan/3/2011
A/duck/Vietnam/LBM132/2012
A/Muscovy duck/Vietnam/LBM113/2012
A/duck/Vietnam/LBM140/2012
A/duck/Vietnam/LBM139/2012
A/magpie robin/Hong Kong/1897/2008
A/magpie robin/Hong Kong/1097/2008
A/grey heron/Hong Kong/1046/2008
A/grey heron/Hong Kong/3088/2007
A/great egret/Hong Kong/807/2008
A/peregrine falcon/Hong Kong/1143/2007
South Korea/Japan
2010-2011
A/Daurian starling/Hong Kong/1532/2007
A/water/Hunan/7/2009
A/swan/Shanghai/10/2009
A/black crowned night heron/Hong Kong/659/2008
A/duck/Guangdong/23/2004
A/silky chicken/Shantou/475/2004
A/duck/China/E319 2/03
A/chicken/Guangdong/178/04
A/chicken/Guangdong/1/2005
A/pika/Qinghai/SHK/2007
A/pika/Qinghai/HMH/2007
A/pika/Qinghai/GRL/2007
A/pika/Qinghai/QW/2007
A/Muscovy duck/Vietnam/1455/2006
A/Owstons civet/Vietnam/1/2005
A/duck/Viet Nam/12/2005
A/chicken/Viet Nam/17/2005
A/quail/Viet Nam/15/2005
A/chicken/Viet Nam/10/2005
A/duck/Yunnan/5877/2005
A/duck/Guangxi/89/2006
A/duck/Vietnam/203/2005
A/duck/Vietnam/204/2005
A/duck/Lang Son/201/2005
A/duck/Vietnam/201/2005
A/duck/Vietnam/206/2005
Indonesian
Sept – Nov 2012
0.005

C

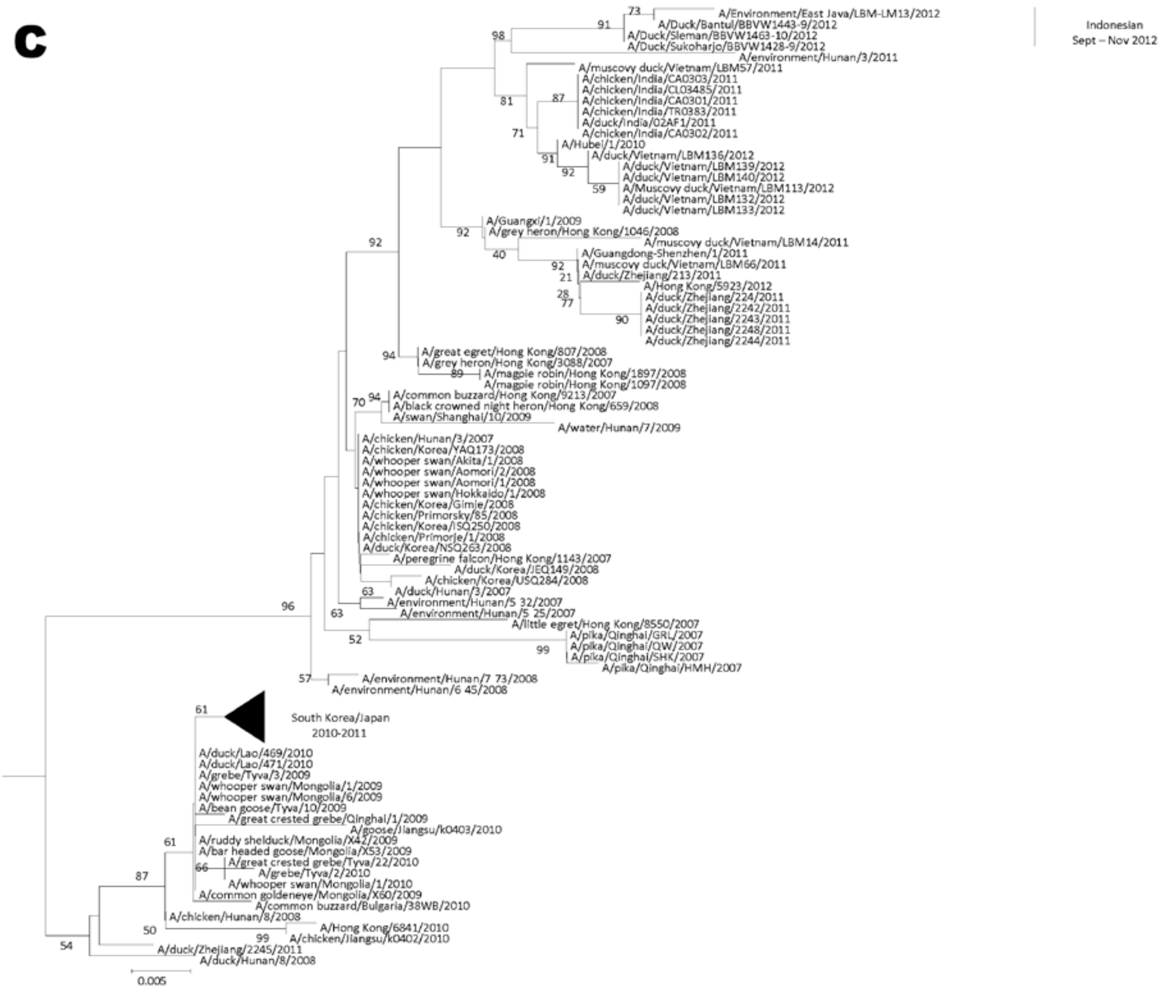
A/Environment/East Java/LBM-LM13/2012
A/Duck/Bantul/BBVW1443-9/2012
A/Duck/Sleman/BBVW1463-10/2012
A/Duck/Sukoharjo/BBVW1428-9/2012
Indonesian
Sept – Nov 2012
A/environment/Hunan/3/2011
A/muscovy duck/Vietnam/LBM57/2011
A/chicken/India/CA0303/2011
A/chicken/India/CL03485/2011
A/chicken/India/CA0301/2011
A/chicken/India/TR0383/2011
A/duck/India/02AF1/2011
A/chicken/India/CA0302/2011
A/Hubei/1/2010
A/duck/Vietnam/LBM136/2012
A/duck/Vietnam/LBM139/2012
A/duck/Vietnam/LBM140/2012
A/Muscovy duck/Vietnam/LBM113/2012
A/duck/Vietnam/LBM132/2012
A/duck/Vietnam/LBM133/2012
A/Guangxi/1/2009
A/grey heron/Hong Kong/1046/2008
A/muscovy duck/Vietnam/LBM14/2011
A/Guangdong-Shenzhen/1/2011
A/muscovy duck/Vietnam/LBM66/2011
A/duck/Zhejiang/213/2011
A/Hong Kong/5923/2012
A/duck/Zhejiang/224/2011
A/duck/Zhejiang/2242/2011
A/duck/Zhejiang/2243/2011
A/duck/Zhejiang/2248/2011
A/duck/Zhejiang/2244/2011
A/great egret/Hong Kong/807/2008
A/grey heron/Hong Kong/3088/2007
A/magpie robin/Hong Kong/1897/2008
A/magpie robin/Hong Kong/1097/2008
A/common buzzard/Hong Kong/9213/2007
A/black crowned night heron/Hong Kong/659/2008
A/swan/Shanghai/10/2009
A/water/Hunan/7/2009
A/chicken/Hunan/3/2007
A/chicken/Korea/YAQ173/2008
A/whooper swan/Akita/1/2008
A/whooper swan/Aomori/2/2008
A/whooper swan/Aomori/1/2008
A/whooper swan/Hokkaido/1/2008
A/chicken/Korea/Gimje/2008
A/chicken/Primorsky/85/2008
A/chicken/Korea/ISQ250/2008
A/chicken/Primorje/1/2008
A/duck/Korea/NSQ263/2008
A/peregrine falcon/Hong Kong/1143/2007
A/duck/Korea/JEQ149/2008
A/chicken/Korea/USQ284/2008
A/duck/Hunan/3/2007
A/environment/Hunan/5 32/2007
A/environment/Hunan/5 25/2007
A/little egret/Hong Kong/8550/2007
A/pika/Qinghai/GRL/2007
A/pika/Qinghai/QW/2007
A/pika/Qinghai/SHK/2007
A/pika/Qinghai/HMH/2007
A/environment/Hunan/7 73/2008
A/environment/Hunan/6 45/2008
South Korea/Japan
2010-2011
A/duck/Lao/469/2010
A/duck/Lao/471/2010
A/grebe/Tyva/3/2009
A/whooper swan/Mongolia/1/2009
A/whooper swan/Mongolia/6/2009
A/bean goose/Tyva/10/2009
A/great crested grebe/Qinghai/1/2009
A/goose/Jiangsu/k0403/2010
A/ruddy shelduck/Mongolia/X42/2009
A/bar headed goose/Mongolia/X53/2009
A/great crested grebe/Tyva/22/2010
A/grebe/Tyva/2/2010
A/whooper swan/Mongolia/1/2010
A/common goldeneye/Mongolia/X60/2009
A/common buzzard/Bulgaria/38WB/2010
A/chicken/Hunan/8/2008
A/Hong Kong/6841/2010
A/chicken/Jiangsu/k0402/2010
A/duck/Zhejiang/2245/2011
A/duck/Hunan/8/2008
0.005

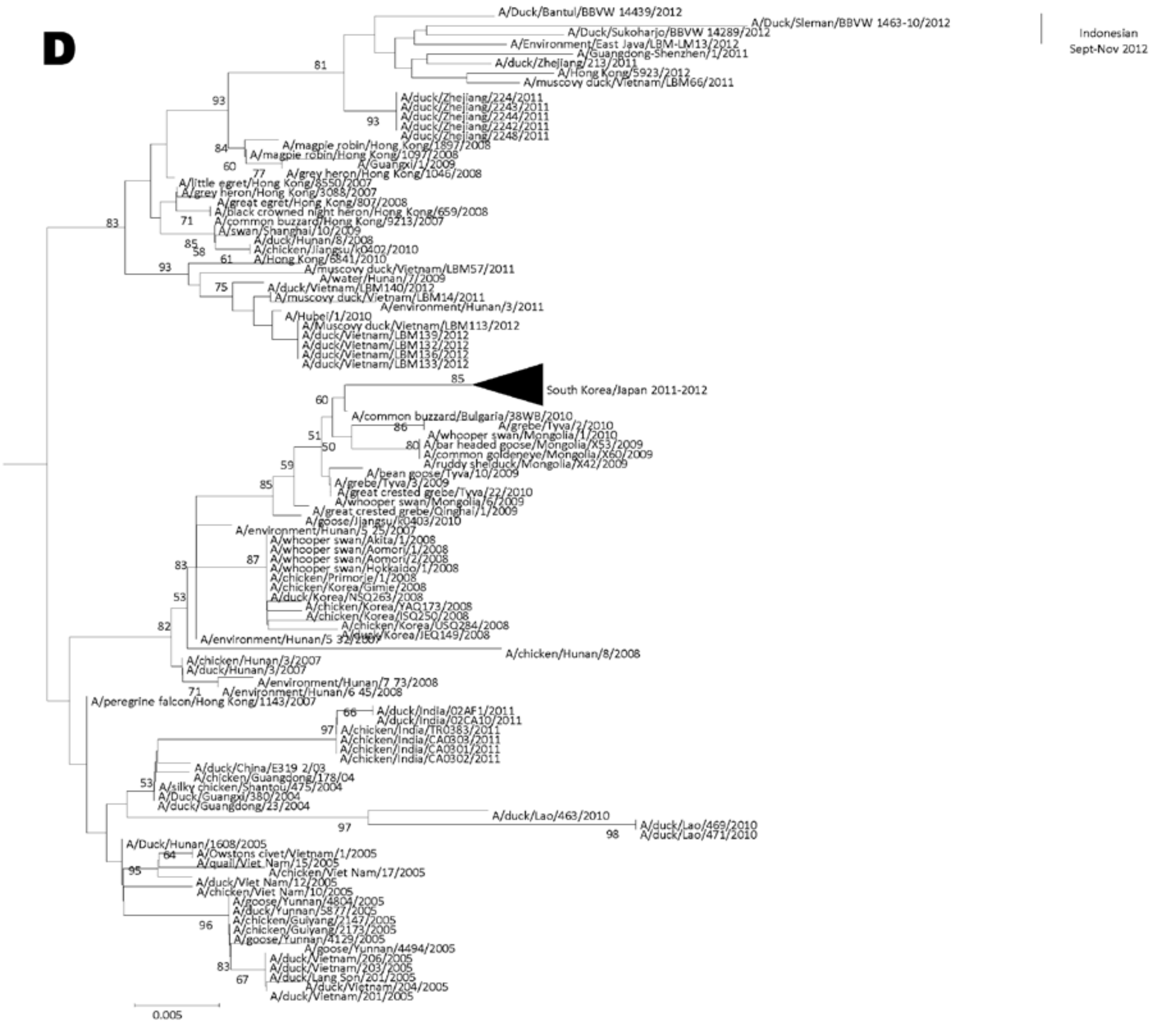
D
A/Duck/Bantul/BBVW 14439/2012
A/Duck/Sleman/BBVW 1463-10/2012
A/Duck/Sukoharjo/BBVW 14289/2012
A/Environment/East Java/LBM-LM13/2012
A/Guangdong-Shenzhen/1/2011
A/duck/Zhejiang/213/2011
A/Hong Kong/5923/2012
A/muscovy duck/Vietnam/LBM66/2011
Indonesian
Sept-Nov 2012
A/duck/Zhejiang/224/2011
A/duck/Zhejiang/2243/2011
A/duck/Zhejiang/2244/2011
A/duck/Zhejiang/2242/2011
A/duck/Zhejiang/2248/2011
A/magpie robin/Hong Kong/1897/2008
A/magpie robin/Hong Kong/1097/2008
A/Guangxi/1/2009
A/grey heron/Hong Kong/1046/2008
A/little egret/Hong Kong/8550/2007
A/grey heron/Hong Kong/3088/2007
A/great egret/Hong Kong/807/2008
A/black crowned night heron/Hong Kong/659/2008
A/common buzzard/Hong Kong/9213/2007
A/swan/Shanghai/10/2009
A/duck/Hunan/8/2008
A/chicken/Jiangsu/k0402/2010
A/Hong Kong/6841/2010
A/muscovy duck/Vietnam/LBM57/2011
A/water/Hunan/7/2009
A/duck/Vietnam/LBM140/2012
A/muscovy duck/Vietnam/LBM14/2011
A/environment/Hunan/3/2011
A/Hubei/1/2010
A/Muscovy duck/Vietnam/LBM113/2012
A/duck/Vietnam/LBM139/2012
A/duck/Vietnam/LBM132/2012
A/duck/Vietnam/LBM136/2012
A/duck/Vietnam/LBM133/2012
South Korea/Japan 2011-2012
A/common buzzard/Bulgaria/38WB/2010
A/grebe/Tyva/2/2010
A/whooper swan/Mongolia/1/2010
A/bar headed goose/Mongolia/X53/2009
A/common goldeneye/Mongolia/X60/2009
A/ruddy shelduck/Mongolia/X42/2009
A/bean goose/Tyva/10/2009
A/grebe/Tyva/3/2009
A/great crested grebe/Tyva/22/2010
A/whooper swan/Mongolia/6/2009
A/great crested grebe/Qinghai/1/2009
A/goose/Jiangsu/k0403/2010
A/environment/Hunan/5 25/2007
A/whooper swan/Akita/1/2008
A/whooper swan/Aomori/1/2008
A/whooper swan/Aomori/2/2008
A/whooper swan/Hokkaido/1/2008
A/chicken/Primorje/1/2008
A/chicken/Korea/Gimje/2008
A/duck/Korea/NSQ263/2008
A/chicken/Korea/YAQ173/2008
A/chicken/Korea/ISQ250/2008
A/chicken/Korea/USQ284/2008
A/duck/Korea/JEQ149/2008
A/environment/Hunan/5 32/2007
A/chicken/Hunan/8/2008
A/chicken/Hunan/3/2007
A/duck/Hunan/3/2007
A/environment/Hunan/7 73/2008
A/environment/Hunan/6 45/2008
A/peregrine falcon/Hong Kong/1143/2007
A/duck/India/02AF1/2011
A/duck/India/02CA10/2011
A/chicken/India/TR0383/2011
A/chicken/India/CA0303/2011
A/chicken/India/CA0301/2011
A/chicken/India/CA0302/2011
A/duck/China/E319 2/03
A/chicken/Guangdong/178/04
A/silky chicken/Shantou/475/2004
A/Duck/Guangxi/380/2004
A/duck/Guangdong/23/2004
A/duck/Lao/463/2010
A/duck/Lao/469/2010
A/duck/Lao/471/2010
A/Duck/Hunan/1608/2005
A/Owstons civet/Vietnam/1/2005
A/quail/Viet Nam/15/2005
A/chicken/Viet Nam/17/2005
A/duck/Viet Nam/12/2005
A/chicken/Viet Nam/10/2005
A/goose/Yunnan/4804/2005
A/duck/Yunnan/5877/2005
A/chicken/Guiyang/2147/2005
A/chicken/Guiyang/2173/2005
A/goose/Yunnan/4129/2005
A/goose/Yunnan/4494/2005
A/duck/Vietnam/206/2005
A/duck/Vietnam/203/2005
A/duck/Lang Son/201/2005
A/duck/Vietnam/204/2005
A/duck/Vietnam/201/2005
0.005

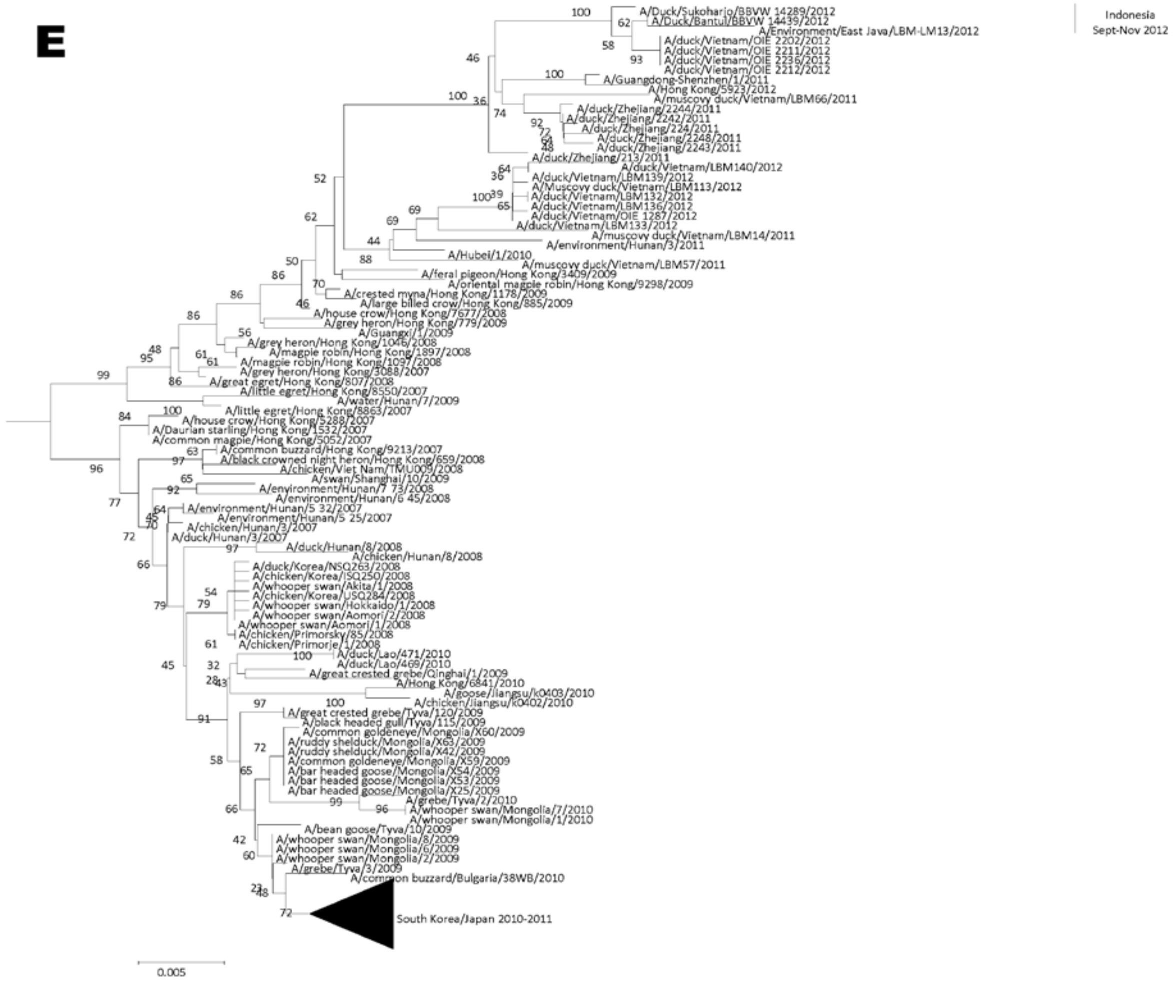
E
A/Duck/Sukoharjo/BBVW 14289/2012
A/Duck/Bantul/BBVW 14439/2012
A/Environment/East Java/LBM-LM13/2012
A/duck/Vietnam/OIE 2202/2012
A/duck/Vietnam/OIE 2211/2012
A/duck/Vietnam/OIE 2236/2012
A/duck/Vietnam/OIE 2212/2012
Indonesia
Sept-Nov 2012
A/Guangdong-Shenzhen/1/2011
A/Hong Kong/5923/2012
A/muscovy duck/Vietnam/LBM66/2011
A/duck/Zhejiang/2244/2011
A/duck/Zhejiang/2242/2011
A/duck/Zhejiang/224/2011
A/duck/Zhejiang/2248/2011
A/duck/Zhejiang/2243/2011
A/duck/Zhejiang/213/2011
A/duck/Vietnam/LBM140/2012
A/duck/Vietnam/LBM139/2012
A/Muscovy duck/Vietnam/LBM113/2012
A/duck/Vietnam/LBM132/2012
A/duck/Vietnam/LBM136/2012
A/duck/Vietnam/OIE 1287/2012
A/duck/Vietnam/LBM133/2012
A/muscovy duck/Vietnam/LBM14/2011
A/environment/Hunan/3/2011
A/Hubei/1/2010
A/muscovy duck/Vietnam/LBM57/2011
A/feral pigeon/Hong Kong/3409/2009
A/oriental magpie robin/Hong Kong/9298/2009
A/crested myna/Hong Kong/1178/2009
A/large billed crow/Hong Kong/885/2009
A/house crow/Hong Kong/7677/2008
A/grey heron/Hong Kong/779/2009
A/Guangxi/1/2009
A/grey heron/Hong Kong/1046/2008
A/magpie robin/Hong Kong/1897/2008
A/magpie robin/Hong Kong/1097/2008
A/grey heron/Hong Kong/3088/2007
A/great egret/Hong Kong/807/2008
A/little egret/Hong Kong/8550/2007
A/water/Hunan/7/2009
A/little egret/Hong Kong/8863/2007
A/house crow/Hong Kong/5288/2007
A/Daurian starling/Hong Kong/1532/2007
A/common magpie/Hong Kong/5052/2007
A/common buzzard/Hong Kong/9213/2007
A/black crowned night heron/Hong Kong/659/2008
A/chicken/Viet Nam/TMU009/2008
A/swan/Shanghai/10/2009
A/environment/Hunan/7 73/2008
A/environment/Hunan/6 45/2008
A/environment/Hunan/5 32/2007
A/environment/Hunan/5 25/2007
A/chicken/Hunan/3/2007
A/duck/Hunan/3/2007
A/duck/Hunan/8/2008
A/chicken/Hunan/8/2008
A/duck/Korea/NSQ263/2008
A/chicken/Korea/ISQ250/2008
A/whooper swan/Akita/1/2008
A/chicken/Korea/USQ284/2008
A/whooper swan/Hokkaido/1/2008
A/whooper swan/Aomori/2/2008
A/whooper swan/Aomori/1/2008
A/chicken/Primorsky/85/2008
A/chicken/Primorje/1/2008
A/duck/Lao/471/2010
A/duck/Lao/469/2010
A/great crested grebe/Qinghai/1/2009
A/Hong Kong/6841/2010
A/goose/Jiangsu/k0403/2010
A/chicken/Jiangsu/k0402/2010
A/great crested grebe/Tyva/120/2009
A/black headed gull/Tyva/115/2009
A/common goldeneye/Mongolia/X60/2009
A/ruddy shelduck/Mongolia/X63/2009
A/ruddy shelduck/Mongolia/X42/2009
A/common goldeneye/Mongolia/X59/2009
A/bar headed goose/Mongolia/X54/2009
A/bar headed goose/Mongolia/X53/2009
A/bar headed goose/Mongolia/X25/2009
A/grebe/Tyva/2/2010
A/whooper swan/Mongolia/7/2010
A/whooper swan/Mongolia/1/2010
A/bean goose/Tyva/10/2009
A/whooper swan/Mongolia/8/2009
A/whooper swan/Mongolia/6/2009
A/whooper swan/Mongolia/2/2009
A/grebe/Tyva/3/2009
A/common buzzard/Bulgaria/38WB/2010
South Korea/Japan 2010-2011
0.005

F

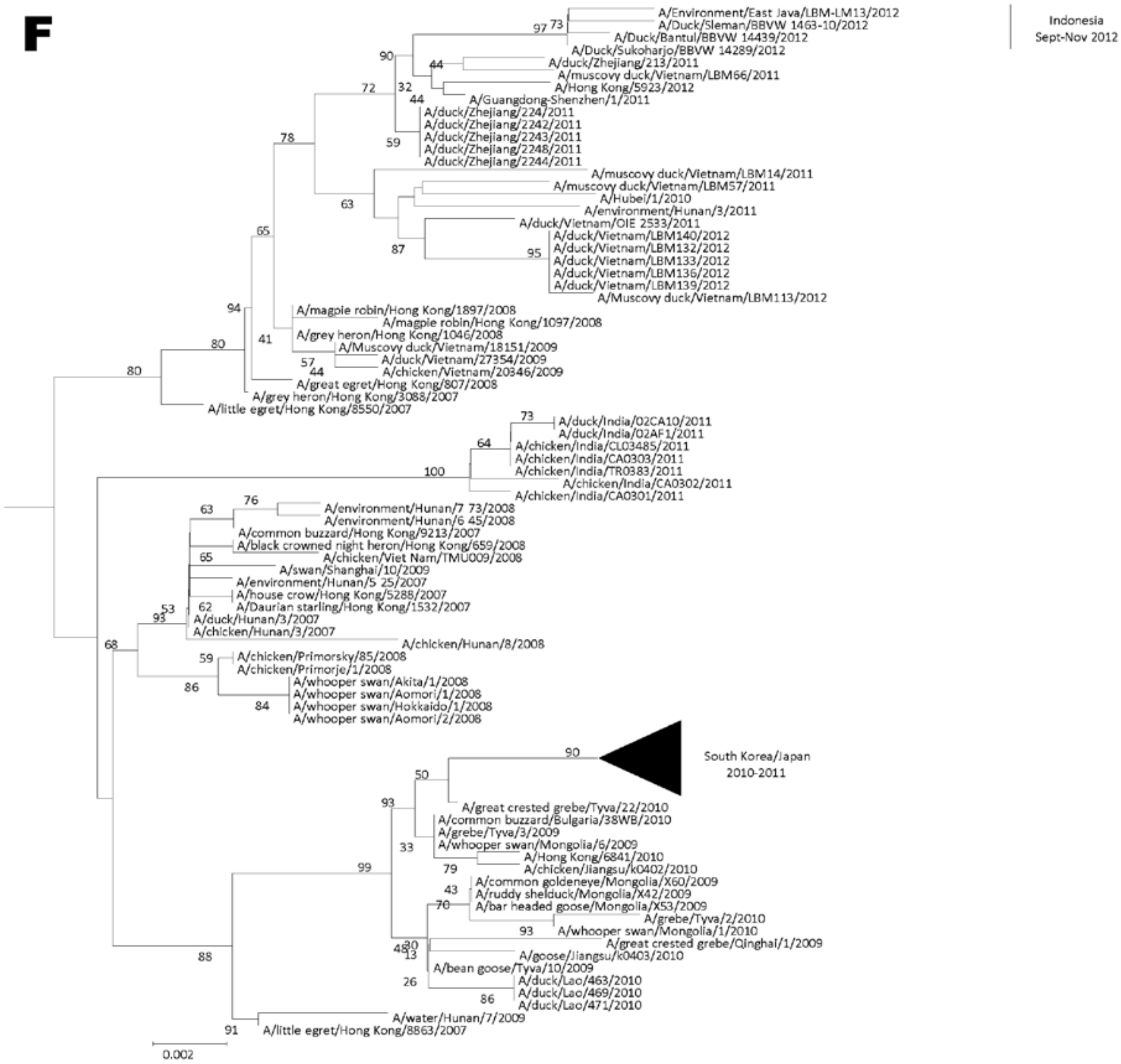
A/Environment/East Java/LBM-LM13/2012
A/Duck/Sleman/BBVW 1463-10/2012
A/Duck/Bantul/BBVW 14439/2012
A/Duck/Sukoharjo/BBVW 14289/2012
Indonesia
Sept-Nov 2012
A/duck/Zhejiang/213/2011
A/muscovy duck/Vietnam/LBM66/2011
A/Hong Kong/5923/2012
A/Guangdong-Shenzhen/1/2011
A/duck/Zhejiang/224/2011
A/duck/Zhejiang/2242/2011
A/duck/Zhejiang/2243/2011
A/duck/Zhejiang/2248/2011
A/duck/Zhejiang/2244/2011
A/muscovy duck/Vietnam/LBM14/2011
A/muscovy duck/Vietnam/LBM57/2011
A/Hubei/1/2010
A/environment/Hunan/3/2011
A/duck/Vietnam/OIE 2533/2011
A/duck/Vietnam/LBM140/2012
A/duck/Vietnam/LBM132/2012
A/duck/Vietnam/LBM133/2012
A/duck/Vietnam/LBM136/2012
A/duck/Vietnam/LBM139/2012
A/Muscovy duck/Vietnam/LBM113/2012
A/magpie robin/Hong Kong/1897/2008
A/magpie robin/Hong Kong/1097/2008
A/grey heron/Hong Kong/1046/2008
A/Muscovy duck/Vietnam/18151/2009
A/duck/Vietnam/27354/2009
A/chicken/Vietnam/20346/2009
A/great egret/Hong Kong/807/2008
A/grey heron/Hong Kong/3088/2007
A/little egret/Hong Kong/8550/2007
A/duck/India/02CA10/2011
A/duck/India/02AF1/2011
A/chicken/India/CL03485/2011
A/chicken/India/CA0303/2011
A/chicken/India/TR0383/2011
A/chicken/India/CA0302/2011
A/chicken/India/CA0301/2011
A/environment/Hunan/7 73/2008
A/environment/Hunan/6 45/2008
A/common buzzard/Hong Kong/9213/2007
A/black crowned night heron/Hong Kong/659/2008
A/chicken/Viet Nam/TMU009/2008
A/swan/Shanghai/10/2009
A/environment/Hunan/5 25/2007
A/house crow/Hong Kong/5288/2007
A/Daurian starling/Hong Kong/1532/2007
A/duck/Hunan/3/2007
A/chicken/Hunan/3/2007
A/chicken/Hunan/8/2008
A/chicken/Primorsky/85/2008
A/chicken/Primorje/1/2008
A/whooper swan/Akita/1/2008
A/whooper swan/Aomori/1/2008
A/whooper swan/Hokkaido/1/2008
A/whooper swan/Aomori/2/2008
South Korea/Japan
2010-2011
A/great crested grebe/Tyva/22/2010
A/common buzzard/Bulgaria/38WB/2010
A/grebe/Tyva/3/2009
A/whooper swan/Mongolia/6/2009
A/Hong Kong/6841/2010
A/chicken/Jiangsu/k0402/2010
A/common goldeneye/Mongolia/X60/2009
A/ruddy shelduck/Mongolia/X42/2009
A/bar headed goose/Mongolia/X53/2009
A/grebe/Tyva/2/2010
A/whooper swan/Mongolia/1/2010
A/great crested grebe/Qinghai/1/2009
A/goose/Jiangsu/k0403/2010
A/bean goose/Tyva/10/2009
A/duck/Lao/463/2010
A/duck/Lao/469/2010
A/duck/Lao/471/2010
A/water/Hunan/7/2009
A/little egret/Hong Kong/8863/2007
0.002

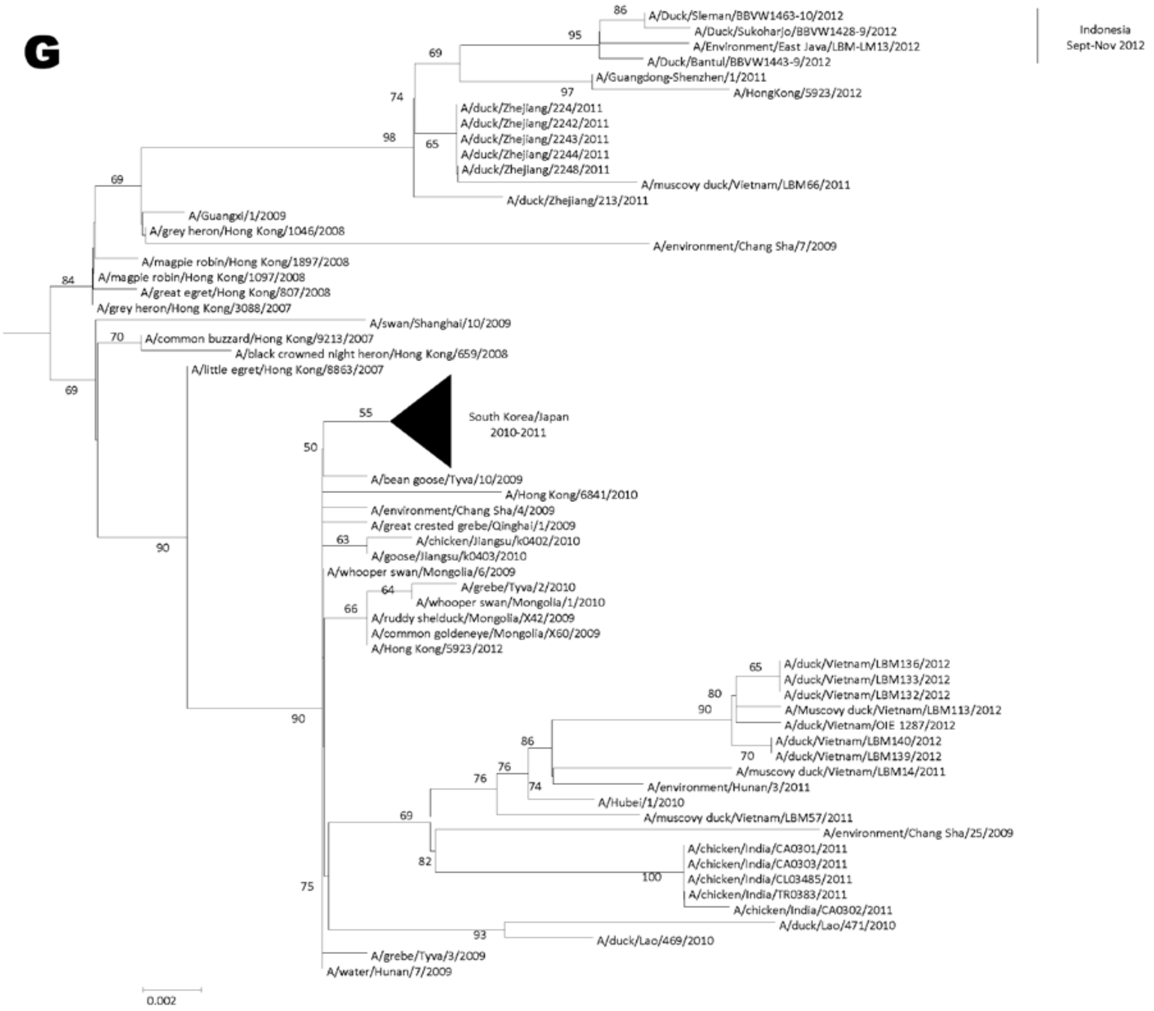

Technical Appendix 1, A–G. Phylogenetic tree of PB2, PB1, PA, NP, NA, M, NS (A = PB2; B = PB1; C = PA; D = NP; E = NA; F = M; G = NS). The phylogenetic tree was generated in MEGA version 4, using neighbor-joining analysis with 1000 bootstrap replicates using the Kimura-2 parameter model. Viruses characterized in this study are indicated with a bar showing samples collected in Indonesia from September to November 2012.
